# Impact of variable titer COVID-19 convalescent plasma and recipient SARS-CoV2-specific humoral immunity on survival in hospitalized patients

**DOI:** 10.1371/journal.pone.0309449

**Published:** 2024-10-24

**Authors:** Carlo J. Iasella, Stefanie J. Hannan, Emily J. Lyons, Sophia C. Lieber, Antu Das, Dimiter Dimitrov, Wei Li, Melissa Saul, Iulia Popescu, Ritchie Koshy, Robin Burke, Braidon Lape, Mark J. Brown, Xiaoping Chen, John C. Sembrat, Kaitlyn Devonshire, Georgios D. Kitsios, Ioannis Konstantinidis, Mark E. Snyder, Bill B. Chen, Christian A. Merlo, David N. Hager, Joseph E. Kiss, Mark H. Yazer, Alan H. Wells, Alison Morris, Bryan J. McVerry, Deborah K. McMahon, Darrell J. Triulzi, John F. McDyer

**Affiliations:** 1 Department of Pharmacy and Therapeutics, University of Pittsburgh School of Pharmacy, Pittsburgh, Pennsylvania, United States of America; 2 Department of Medicine, Division of Pulmonary, Allergy, Critical Care and Sleep Medicine, University of Pittsburgh School of Medicine, Pittsburgh, Pennsylvania, United States of America; 3 Department of Medicine, Aging Institute, University of Pittsburgh School of Medicine, Pittsburgh, Pennsylvania, United States of America; 4 Department of Medicine, Division of Infectious Diseases, University of Pittsburgh School of Medicine, Pittsburgh, Pennsylvania, United States of America; 5 Division of Pulmonary and Critical Care Medicine, Johns Hopkins University School of Medicine, Baltimore, MD, United States of America; 6 Department of Pathology, Division of Transfusion Medicine, University of Pittsburgh School of Medicine, Pittsburgh, Pennsylvania, United States of America; 7 Department of Pathology, Division of Laboratory Medicine, University of Pittsburgh School of Medicine, Pittsburgh, Pennsylvania, United States of America; Consejo Nacional de Investigaciones Cientificas y Tecnicas, ARGENTINA

## Abstract

COVID-19 convalescent plasma (CCP) was one of the first therapies to receive emergency use authorization for management of COVID-19. We assessed the effectiveness of CCP in a propensity-matched analysis, and whether the presence of antibodies in the recipient at the time of treatment or the titer of antibodies in the administered CCP influenced clinical effectiveness. In an inpatient population within a single large health system, a total of 290 CCP patients were matched to 290 controls. While CCP increased titers of anti-SARS-CoV-2 RBD IgG titers post-CCP (p = <0.0001), no differences in 30-day survival were observed between CCP patients and controls in univariate and multivariate analyses. Survival at 30 days was numerically lower in recipients who were seronegative prior to CCP administration, compared to those with low titer and high titer anti-SARS-CoV-2 RBD IgG, respectively, but did not reach statistical significance (56% vs 82% vs 75%, p = 0.16). Patients who received 2 units of high-titer CCP had numerically better survival versus those who received fewer high-titer units, but this was not statistically significant (p = 0.08). CCP did not improve 30-day survival compared to propensity matched controls. Together these data support that CCP therapy provides limited benefit to hospitalized patients with SARS-CoV-2 infection.

## Introduction

Administration of convalescent plasma from survivors of viral outbreaks has been well-described dating back to the early 1900s when it was used during outbreaks of polio, measles, mumps, and influenza [1]. It is thought to benefit patients through provision of pathogen-specific antibodies from previously infected individuals to protect against severe disease and death. It was one of the earliest therapies that received FDA emergency use authorization after the outbreak of COVID-19 in 2020 when other antiviral therapies and monoclonal antibody preparations were unavailable [2].

Convalescent plasma offers theoretical advantages as therapeutic in the setting of infectious outbreaks. Plasma collected in close temporal proximity to prevalent circulating strains should contain antibodies against those strains, mitigating the effect of inevitable mutations that can undermine the efficacy of mass-produced monoclonal antibody therapies. Additionally, plasma is frequently administered in clinical practice and the adverse effect profiles are well understood and generally of minor concern.

Experiences evaluating the use of COVID-19 convalescent plasma (CCP) have had mixed results. Most clinical trials investigating its use in hospitalized populations have not demonstrated benefit [3–6]. CCP has demonstrated mixed results in outpatients, with use early in the disease course showing reduction in the risk of hospitalization or disease progression [7,8], while others showed no benefit [9–11]. Few studies have evaluated the baseline antibody titers of CCP recipients (before CCP administration) or titers in the administered CCP product. The purpose of this study was to evaluate the effectiveness of CCP administration by comparing CCP recipients versus propensity-matched controls in an inpatient population with moderate to severe COVID-19. We also sought to evaluate whether the presence of recipient antibodies before CCP administration and the titer of antibodies in the administered CCP influenced its clinical effectiveness.

## Materials and methods

### Study design

This single health-system, propensity-matched cohort study compared patients who received CCP to those who did not. It was approved by the University of Pittsburgh Institutional Review Board. Clinical information for CCP recipients and controls was extracted from an electronic medical record data repository that contains full-text medical records and integrates information from central transcription, pharmacy, laboratory, finance, administrative, and other departmental databases.

### Patient population

Patients hospitalized within the UPMC system with a diagnosis code for COVID-19 from March 2020 to June 2021 were eligible for inclusion. Patients were excluded if they were less than 18 years of age. Patients were identified in accordance with the National COVID Cohort Collaborative guidance using the presence of a positive COVID-19 test, or an ICD-10 code of U07.1, or two or more COVID-like diagnosis codes during the same encounter prior to 5/1/2020 [12]. Treatment decisions outside of CCP use were made at the discretion of the treating provider.

### Study procedures

Patients were grouped by whether they received CCP. Two units of CCP were administered to participants per protocol. Patients were eligible to receive CCP under Emergency Use Authorization if they met criteria as outlined by the FDA in August of 2020 [2]. For patients who received CCP, prospectively collected blood samples were obtained prior to and 48–72 hours after administration of CCP when possible. Pittsburgh CCP collective was established in collaboration with the local blood collector (Vitalant Northeast, Pittsburgh, PA), UPMC, and Allegheny Health Network that screened individuals with mild infection at least 21 days after resolution of their signs and symptoms for eligibility to donate CCP. Information about this donor population is provided in S1 Table in S1 File. All patient who received CCP were treated between April 2020 and February 2021, before the recommendation of using only high-titer donor CPP was published (FDA Clinical Memorandum Re: EUA 26382A, Feb. 4, 2021).

### Antibody evaluation

As possible, plasma samples pre-and post-CCP transfusion were obtained for analysis in the CCP group for analysis of pre- and post-CCP antibody titers from 10 April 2020 through 30 June 2021. These patients were provided verbal and written informed consent either, which was documented in writing or electronically. Previous studies have shown that antibodies specific to the S1 receptor binding domain (RBD) are among the most potent neutralizing antibodies [13–15]. As such, we developed a traditional sandwich-style ELISA to measure SARS-CoV-2 IgG against the Spike-1 (S1) receptor binding domain (RBD) protein. Plates were coated with RBD, incubated with plasma samples from patients pre- and post-CCP treatment, and analyzed via spectrophotometry at 450 nm wavelength. For donor CCP, titers were obtained after the Orothos Vitros total Ig assay was adopted to screen product. The S/C ratio for antibody titer was obtained using the Ortho Vitros total Ig assay, performed at the Vitalant laboratory. Values > 20 were considered to be positive while those > 200 were characterized as high-titer CCP units, in accordance with FDA guidance [2].

### Study outcomes

Clinical information including patient demographics, hospital visit information, diagnosis and procedure codes, medication charges, degree of respiratory support, and survival information were extracted from the medical record by the study team for both CCP recipients and controls. Data collection by the study team was completed on 30 April 2022. Respiratory support was stratified by level of support (i.e., none, oxygen requirement, mechanical ventilation, and extracorporeal membrane oxygenation [ECMO]). Additional clinical variables including CCP administration time were collected for the CCP group.

The primary outcome was patient survival at 30 days obtained from the hospital discharge disposition and the Social Security Death Index. Additional outcomes of interest included survival to hospital discharge, length of stay, and duration of mechanical ventilation or ECMO. Outcomes were assessed between CCP and control groups, stratified by timing of CCP administration (prior to or after 72 hours of admission), and baseline antibody serostatus. Additionally, we completed subgroup analyses within the CCP cohort to assess the effects of baseline SARS-CoV-2 antibody titer and CCP titer on 30-day survival.

### Statistical analysis

CCP recipients and controls were matched 1:1 using propensity matching techniques with the package MatchIt (Ho DE (2011)) for R (R Core Team (2022); Vienna, Austria) including age, sex, race, and level of respiratory support We included month of participation in the matching criteria because the availability of COVID-19 treatments changed over time. Continuous variables were assessed using the Mann-Whitney U test or Wilcoxon signed rank test, and categorical variables were assessed using Chi-squared test or Fisher’s Exact Test. Time to event analyses were completed using Kaplan-Meier methods and multivariable Cox Proportional Hazards models. For the multivariable analyses, variables were eligible for inclusion in the final model if their univariable p-value < 0.2.

## Results

**Fig 1:**
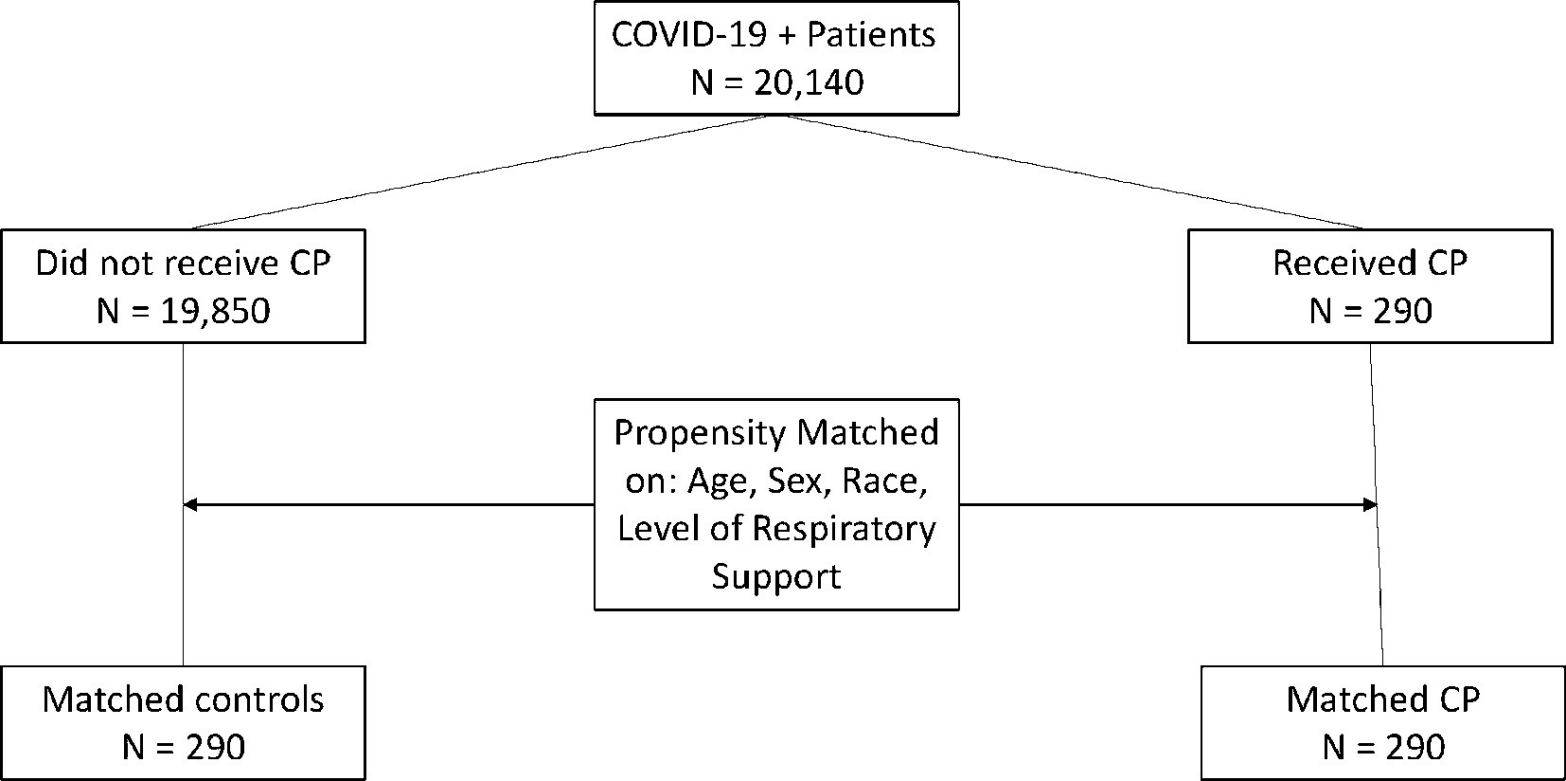
Consort diagram showing study cohort.

A total of 20,140 patients who had hospital or emergency department visits for COVID-19 during the study period (Fig 1). Of these, 290 patients received CCP, leaving a control pool of 19,850 patients for matching. After matching, the CCP and control cohorts were similar in age, sex, race, month of hospitalization, and level of respiratory support (Table 1). For patients who received CCP treatment, the median time to transfusion was 3.5 days from admission (IQR: 2–6 days).

**Table 1:** Patient characteristics.

	Unmatched Cohort			Matched Cohort		
	Control (n = 19,850)	CCP (n = 290)	P-value	Control (n = 290)	CCP (n = 290)	P-value
Age (median, IQR)	62 [45–75]	67 [57–75]	<0.001	68 [58–77]	67 [57–75]	0.38
Female	10,733 (54.1%)	108 (37.2%)	<0.001	96 (33.1%)	108 (37.2%)	0.30
Race			0.02			0.48
White	16,071 (81%)	254 (88%)		261 (90%)	254 (88%)	
Black	2885 (14%)	24 (8%)		22 (8%)	24 (8%)	
Other	360 (2%)	4 (1%)		4 (1%)	4 (1%)	
Declined	534 (3%)	8 (3%)		3(1%)	8 (3%)	
Respiratory Support			<0.001			0.98
None	12,350 (62%)	17 (6%)		18 (6%)	17 (6%)	
Oxygen/Non-invasive	5638 (28%)	118 (41%)		118 (41%)	118 (41%)	
MV/ECMO	1862 (9%)	155 (53%)		154 (53%)	155 (53%)	

**Fig 2:**
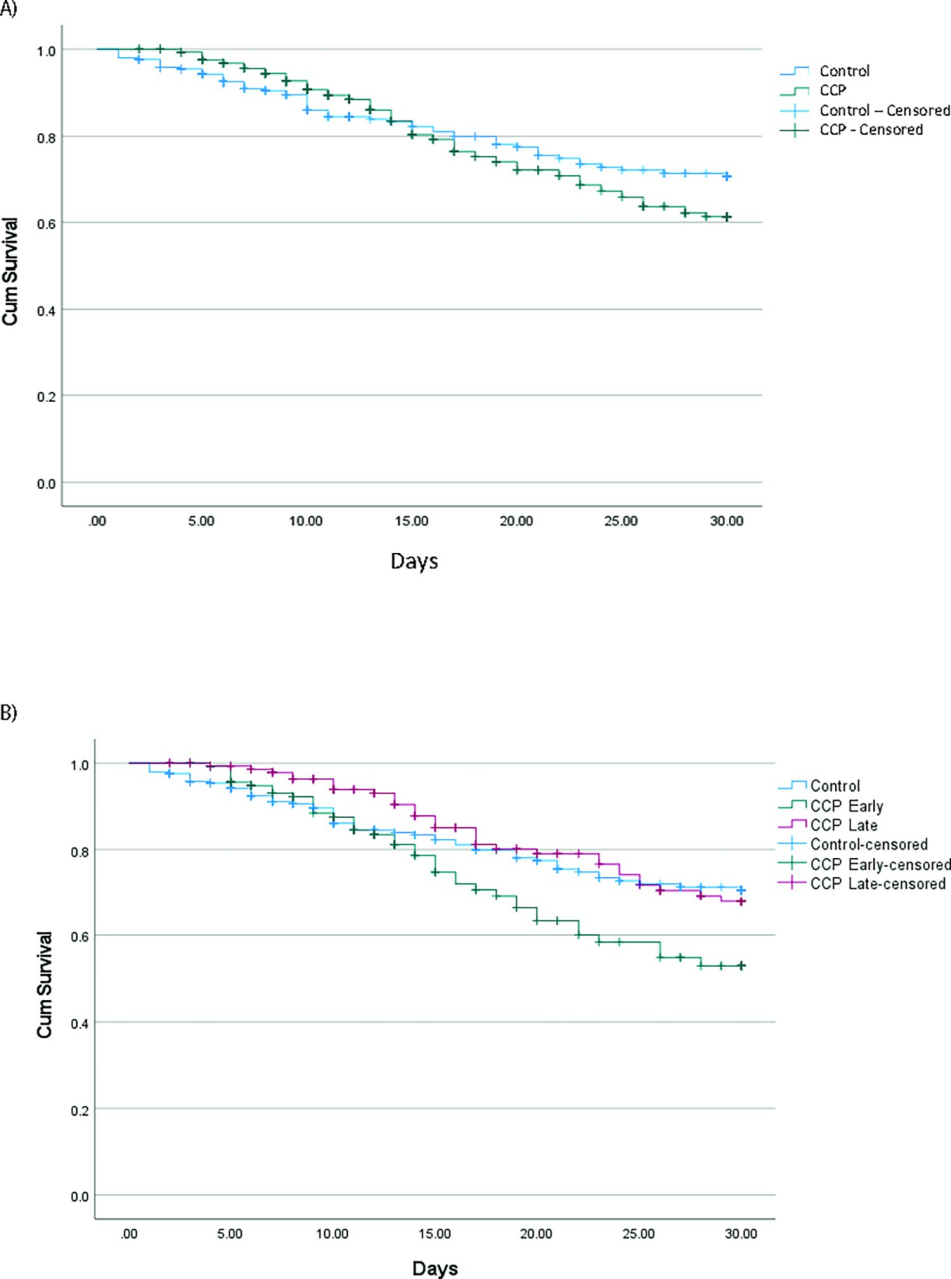
A) Overall 30-day survival in CCP recipients versus controls; B) 30-day survival in cohort adjust for early CCP therapy (within 72 h) or late CCP therapy. NS = not significant.

In the matched cohort, overall survival at 30 days was 77.4%. There was no significant difference in 30-day mortality between CPP and control groups groups (25% vs 20%, p = 0.14) (Fig 2). This finding was maintained on multivariable Cox regression controlling for age, sex, and respiratory support. No differences were observed when stratifying patients by mechanical ventilation status or ECMO, or between CP recipients who received CP within 72 hours versus later (S2 Table in S1 File). No difference in survival was observed between CCP non-recipients, those who received CCP within 72 hours of admission and those who received it later after admission. These findings were maintained on multivariable Cox Proportional Hazards modeling (S2 Table in S1 File). No differences were observed in hospital length of stay or duration of mechanical ventilation or ECMO between the CCP and control groups.

**Fig 3:**
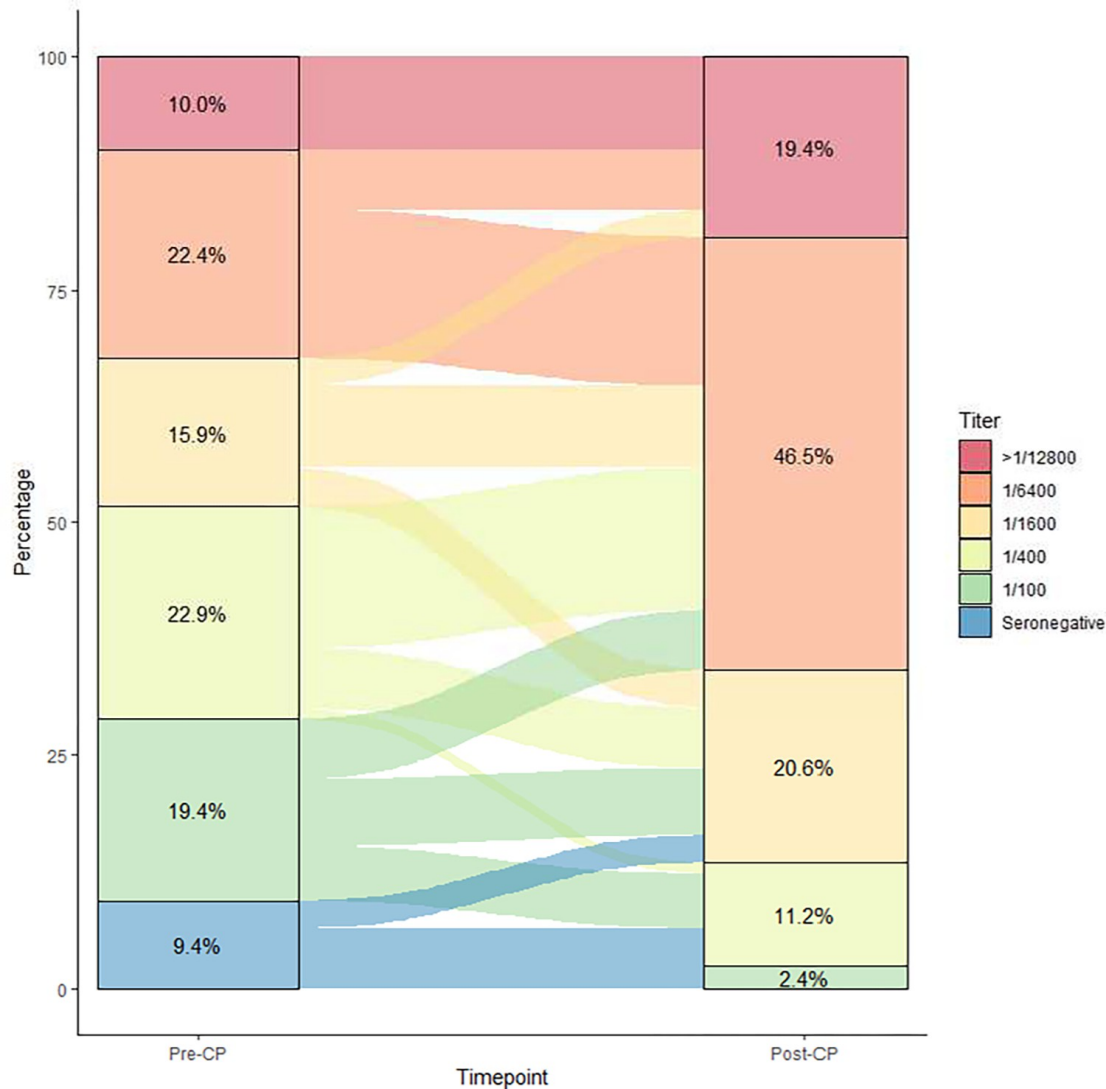
Sankey plot showing pre-CCP and post-CCP anti-RMD titers as determined by ELISA.

Plasma samples before and after receipt of CPP were available for 175/290 (60%) patients. Among the pre-CCP transfusion samples, 9.1% (n = 16) had nondetectable amounts of RBD IgG and 18.9% (n = 33) had a titer of 100 (low titer) (Fig 3). Most patients, 71.84% (n = 126), had detectable titers pre-CCP therapy of 400 or greater (high titer) at baseline. Among the 58 patients who had pre-CCP titers at 6400, 19 of these had a titer of 12,800 or more. Overall, patients were found to have a statistically significant increase in anti-SARS-CoV-2 RBD IgG titers post-CCP administration (p = <0.0001). Group changes before and after CCP administration are shown in Fig 3.

CCP patients were stratified into three groups by seropositivity for SARS-CoV-2 antibody prior to receipt of CCP: seronegative, low titer defined as detectable antibody at 100 but absent at 400, or high titer defined as detectable antibody at 1/400 or greater. No significant differences were observed in 30-day survival or survival to hospital discharge between groups, though seronegative trended towards lower survival (56%) compared to low baseline titer (82%) and high baseline titer (75%; p = 0.16). These findings were maintained after adjustment in multivariable Cox Proportional Hazards models. Only increasing age and mechanical ventilation or ECMO were significantly associated with an increased hazard of death. We performed sensitivity analyses stratifying classifying patients at baseline as seronegative or positive at titers of 100, 400, 1600, or ≥ 6400 with similar results.

**Fig 4:**
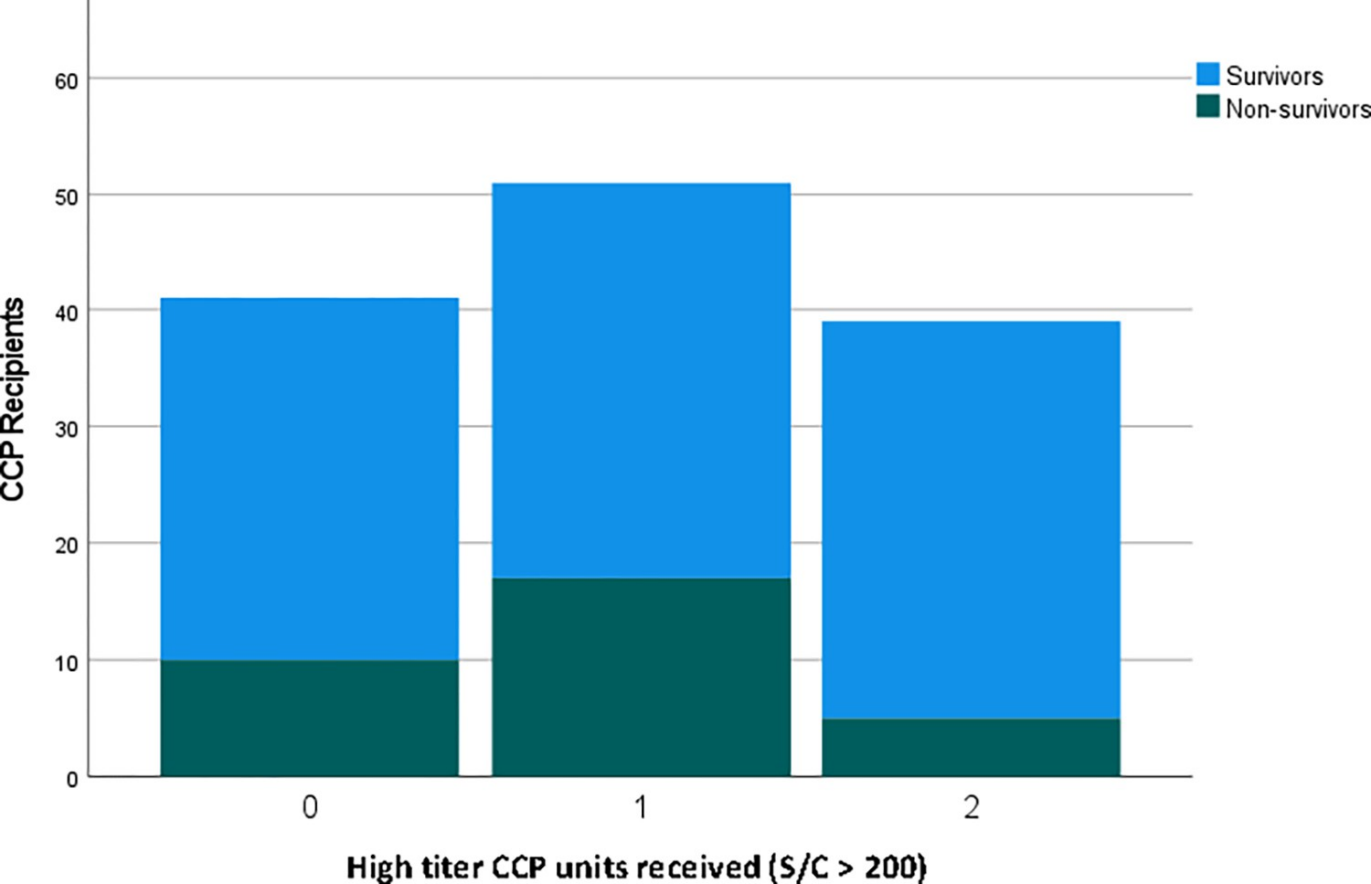
Comparison of survival according to administration of high titer CCP units.

Information regarding donor CCP concentration was available for both plasma units in 133 CCP recipients (45%). While all patients received two units of CCP, 42 (31.6%) received zero units of high titer CCP, 51 (38.3%) received 1 unit of high titer CCP, and 40 (30.1%) received 2 units of high titer CCP (Fig 4). Those who received 2 units of high titer CCP had the highest rate of 30-day survival at 87.2% versus 75.6% in the zero units group and 66.7% in the 1 unit group, though this did not reach statistical significance (p = 0.08). A Cox Proportion Hazards model including donor CCP concentration and recipient antibody titer maintained these findings (Table 2).

**Table 2:** Cox Proportional Hazards model for survival at 30 days among CCP recipients.

Variable	Hazard Ratio	95% CI	p-value
Age (years)	1.10	1.05–1.17	<0.001
Recipient seronegative	Comparator	-	-
Recipient low titer	0.16	0.04–0.74	0.02
Recipient high titer	0.33	0.11–1.03	0.06
Donor units both low titer	Comparator	-	-
Donor units one high titer	1.17	0.42–3.29	0.76
Donor units both high titer	0.67	0.18–2.50	0.55

## Discussion

Our study found no significant difference in the overall 30-day survival between those who received CCP and propensity matched controls. While many individual studies of CPP in inpatients have published negative results, a recent large meta-analysis of CCP in hospitalized patients evaluated 39 randomized controlled trials consisting of over 21,000 patients [16]. They observed at 13% reduction in likelihood of mortality for CCP versus controls (OR: 0.87, 95%CI: 0.76–1.00).

Data in outpatient populations has been mixed, with some individual studies showing benefit at preventing disease progression to more severe illness, while others have not [7,17]. A recent meta-analysis of 5 randomized controlled trials in 2620 outpatients found a lower rate of hospitalization in CPP patients vs controls (8.5% vs 12.2%, p = 0.001) [18]. Some differences may be explained by the timing of administration in relationship to their COVID-19 disease course, as has been observed in the use of monoclonal antibodies. Casirivimab/imdevimab reduced the risk of hospitalization and death when used in the outpatient setting in initial trials [19,20]; however the later ACTIV-3 study using the similar monoclonal antibody bamlanivimab to treat hospitalized patients with COVID-19 was stopped early for futility in improving 90-day recovery outcomes [21].

The majority of treated patients in our study were seropositive at the time of receipt of CCP and had a variable increase in their post-infusion antibody levels. Most patients who were low-titer (<100) at baseline saw a significant increase in anti-RBD antibody levels after CCP (400–1600). The smaller population of seronegative patients saw an increase in anti-RBD antibodies, but not to a similar degree as the low titer patients. Interestingly, this smaller population of seronegative patients trended towards worse survival. This suggested that CCP treatment was insufficient to rescue hospitalized patients with seronegative status. This was evaluated previously in a subset of the RECOVERY trial [22,23]. While the initial trial found that high-titer CCP did not improve survival in hospitalized COVID-19 patients, a post hoc subgroup analysis showed that those who were seronegative at the time of treatment were more likely to derive benefit. Differences in the patient populations, severity of illness, and other treatments or clinical factors may account for different observations between the RECOVERY group and our study. Additionally, previous randomized controlled studies evaluating the use of CCP in small populations of immunocompromised patients observed no difference in mortality with CCP [24–26].

Our data suggest that patients who received two units of high-titer CCP had higher rates of 30-day survival compared to those who received one or zero units of high titer CCP. Insufficient antibody titer in CCP given to some patients could explain suboptimal responses. Larger numbers would be required to confirm these findings. The RECOVERY and the DAWn-plasma trials did not show survival benefits among inpatients receiving high titer CCP versus controls who did not receive CCP [22,27]. Importantly, these trials administered between 1–5 units of CCP. The CAPSID trial found that in those with severe COVID-19, the subset that received a larger amount of neutralizing antibodies experienced benefit [28]. Of note, the definition of high-titer CCP varied amongst studies; a standardized definition is needed for more direct comparison of study results. Previous studies have measured donor plasma titer and others recipient plasma titers, but we could not find reports where both are characterized.

In our population of hospitalized patients with moderate to severe COVID-19, 70% were seropositive with high titer antibody (>1/250) at baseline. Therefore, the majority of patients were advanced in their infectious course and CCP administration may not have provided significant immunologic benefit. In contrast, approximately 30% had low titer anti-RBD antibody levels or were seronegative. However, these patients did not benefit from CCP administration, which could be partially explained by receipt of low titer CCP (true of half of our units). Future use of convalescent plasma during pandemics should use strategies that ensure higher titer product [29].

A few limitations of this study should be noted. Control COVID-19 cases were identified in accordance with the National COVID Cohort Collaborative; however, this relied on the accuracy of ICD-10 coding. This could have been more challenging early in the pandemic before a COVID-19 specific ICD-10 was defined. While propensity matching was utilized to reduce the risk of confounding, residual confounding may have been present given the nonrandomized nature of the study. We were also limited by the availability of donor titer information for only a subset of CCP as the Ortho total Ig assay was not available at the beginning of the study period. Additionally, donor antibody titers were not universally assessed prior to administration, limiting the number of patients who received high-titer CCP units as recommended by subsequent FDA guidance. This may account for reduced response rates within the CCP cohort. Finally, the risk of Type II error is also of concern, given the relatively small sample size of this single-center study. Subsequent meta-analyses including these results with similar studies may help to further elucidate the effects of CCP and donor and recipient titer through increasing study power.

Among hospitalized individuals with moderate to severe COVID-19, patients who received 2 units of high-titer CCP had numerically improved 30-day survival, however this difference did not reach statistical significance. The effects of recipient serologies at the time CCP administration and CCP antibody titer warrant further investigation to understand their implications for future novel viruses.

## Supporting information

S1 File Table S1: CCP Donor Characteristics; Table S2: Cox Regression: 30-day Survival.
(DOCX)
