# Supplementary material for: Impact of variable titer COVID-19 convalescent plasma and recipient SARS-CoV2-specific humoral immunity on survival in hospitalized patients
Source: PLoS One. 2024 Oct 24;19(10):e0309449. doi: 10.1371/journal.pone.0309449 (PMC11500870; doi:10.1371/journal.pone.0309449)
Supplement: S1 File — Table S1: CCP Donor Characteristics; Table S2: Cox Regression: 30-day Survival. (DOCX) [file pone.0309449.s001.docx]

Table S1: CCP Donor characteristics

|  | Overall N (%) |
| --- | --- |
| Age, median [IQR] | 49 [34-60] |
| Female | 209 (59%) |
| Self-Reported Race |  |
| White | 313 (89.6%) |
| Black | 17 (4.8%) |
| Asian | 3 (0.8%) |
| Other | 6 (1.7%) |
| Multiple race | 2 (0.5%) |
| No Answer/Unknown | 10 (2.9%) |
| Hispanic | 13 (3.7%) |

Table S2: Cox Regressions: 30-day Survival

|  | **Hazard Ratio** | **Confidence Interval** | **p-value** |
| --- | --- | --- | --- |
| *Model 1 – CCP Transfusion Only* | | | |
| CCP Transfusion | 1.09 | 0.77-1.53 | 0.65 |
| Respiratory Support (Comparator = None) |  |  | <0.01 |
| Oxygen | 0.09 | 0.01-0.61 | 0.01 |
| MV/ECMO | 0.43 | 0.27-0.67 | <0.01 |
| Female | 0.67 | 0.46-0.99 | 0.044 |
| Age | 1.05 | 1.03-1.06 | <0.001 |
| *Model 2 – Early CCP vs Late CCP vs No CCP* | | | |
| No CCP (comparator) | - | - | 0.35 |
| Early CCP | 1.083 | 0.71-1.66 | .71 |
| Late CCP | 1.38 | 0.87-2.19 | .18 |
| Respiratory Support (Comparator = None) | - | - | <0.01 |
| Oxygen | 0.09 | 0.01-0.62 | 0.02 |
| MV/ECMO | 0.43 | 0.28-0.68 | <0.01 |
| Female | 0.67 | 0.45-0.99 | 0.04 |
| Age | 1.05 | 1.03-1.06 | <0.01 |
